# Invasive Solid Papillary Carcinoma of the Breast Initially Diagnosed as Invasive Ductal Carcinoma: A Case Report

**DOI:** 10.70352/scrj.cr.26-0355

**Published:** 2026-07-09

**Authors:** Yuki Asaka, Haruhito Kinoshita, Hanae Matsuda, Saeko Henmi, Yuko Kikukawa, Ayako Gose, Mai Nishimoto, Asuka Kochi, Chika Watanabe, Koji Takada, Yukie Tauchi, Kana Ogisawa, Tamami Morisaki, Kenichi Kohashi, Shinichiro Kashiwagi

**Affiliations:** 1 Department of Breast Surgical Oncology, Osaka Metropolitan University Graduate School of Medicine, Osaka, Osaka, Japan; 2 Department of Pathology, Osaka Metropolitan University Graduate School of Medicine, Osaka, Osaka, Japan

**Keywords:** breast cancer, solid papillary carcinoma, invasive ductal carcinoma, core needle biopsy, neuroendocrine differentiation, synaptophysin, case report

## Abstract

**INTRODUCTION:**

Solid papillary carcinoma of the breast is a rare papillary neoplasm with distinctive morphology and frequent neuroendocrine differentiation. However, when an invasive component is sampled in a limited core needle biopsy specimen, its solid and nested architecture may mimic invasive ductal carcinoma. We report a case of invasive solid papillary carcinoma of the breast that was initially diagnosed as invasive ductal carcinoma and was ultimately confirmed by comprehensive histopathological and immunohistochemical evaluation.

**CASE PRESENTATION:**

A 53-year-old woman was referred to our hospital for treatment of right breast cancer detected by screening. Ultrasonography showed a 1.3-cm mass in the upper outer quadrant of the right breast. CT showed no distant metastasis, and breast MRI showed no apparent intraductal extension. The clinical stage was cT1N0M0, stage I. Core needle biopsy at the referring hospital was interpreted as invasive ductal carcinoma. Pathological review at our institution showed relatively uniform epithelial cells arranged in small nests and solid structures. The tumor was strongly positive for estrogen receptor and progesterone receptor, negative for human epidermal growth factor receptor 2, showed a low Ki-67 labeling index of 5%, and was positive for synaptophysin, suggesting invasive solid papillary carcinoma with neuroendocrine differentiation. The patient underwent breast-conserving surgery and sentinel lymph node biopsy. The resected specimen confirmed invasive solid papillary carcinoma. Postoperative Oncotype DX testing showed a recurrence score of 4. Adjuvant chemotherapy was omitted, and the patient received postoperative radiotherapy followed by endocrine therapy with anastrozole. She remains free of recurrence 1 year after surgery.

**CONCLUSIONS:**

Invasive solid papillary carcinoma can mimic invasive ductal carcinoma on core needle biopsy. Careful morphological assessment combined with appropriate immunohistochemical evaluation is essential for an accurate diagnosis. In the present low-risk luminal case, adjuvant chemotherapy would probably not have been indicated even if the lesion had remained classified as invasive ductal carcinoma of no special type; however, preoperative recognition of this special subtype may still be clinically relevant for biopsy planning, axillary staging, surgical margin planning, and consideration of minimally invasive local treatment.

## Abbreviations

CNB
: core needle biopsy
HER2
: human epidermal growth factor receptor 2
RFA
: radiofrequency ablation
RS
: recurrence score
SLNB
: sentinel lymph node biopsy
VAB
: vacuum-assisted biopsy

## INTRODUCTION

Solid papillary carcinoma of the breast is an uncommon papillary neoplasm, representing less than 1% of all breast carcinomas.^1)^ It is usually encountered in elderly or postmenopausal women and shows distinctive histological features, including solid nodular proliferation, delicate fibrovascular cores, low- to intermediate-grade nuclear atypia, and frequent neuroendocrine differentiation.^2)^ Most tumors are estrogen receptor-positive and HER2-negative, and the overall prognosis is considered favorable compared with that of conventional invasive breast carcinoma.^3,4)^

Despite these characteristic features, the diagnosis of invasive solid papillary carcinoma may be difficult in limited biopsy specimens.^5,6)^ In particular, its solid and nested architecture can resemble invasive ductal carcinoma of no special type, while variable loss of myoepithelial markers may further complicate the distinction between *in situ* and invasive components.^5)^ Similar diagnostic difficulties have been described across the spectrum of papillary breast lesions, particularly when only limited tissue is available for evaluation.^7,8)^ Accurate recognition is important because solid papillary carcinoma has distinct clinicopathological features and is generally associated with an indolent clinical course; therefore, misdiagnosis as conventional invasive ductal carcinoma may lead to unnecessary treatment escalation.^6)^ We herein report a case of invasive solid papillary carcinoma of the breast that was initially interpreted as invasive ductal carcinoma on CNB and was ultimately diagnosed by comprehensive histopathological and immunohistochemical evaluation.

## CASE PRESENTATION

**Fig. 1:**
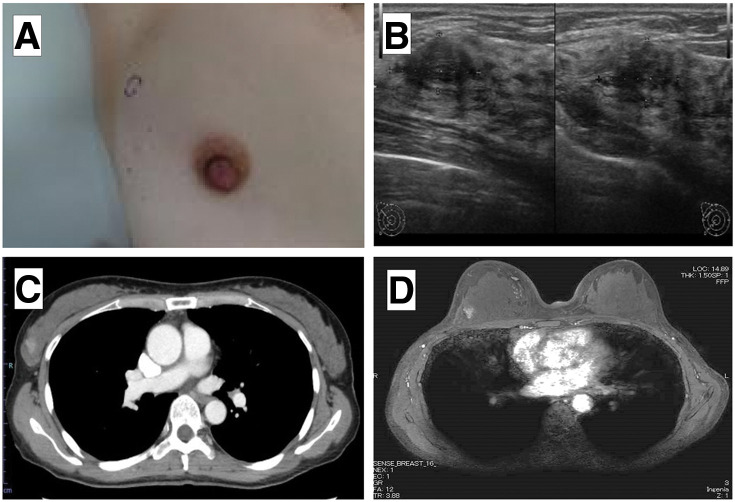
Clinical and imaging findings. (**A**) Physical findings of the right breast at presentation. (**B**) Breast ultrasonography showing a 1.3-cm mass in the upper outer quadrant of the right breast. (**C**) CT showing no evidence of distant metastasis. (**D**) Breast MRI showing no apparent intraductal extension.

A 53-year-old woman was referred to our hospital for treatment of right breast cancer. She had a history of ovarian cyst and no personal history of breast disease. Her father had a history of colorectal cancer. The breast lesion was detected during routine screening, and ultrasonography performed at a previous hospital revealed a mass in the right breast. CNB was initially interpreted as invasive ductal carcinoma, and she was subsequently referred to our institution. Physical findings of the right breast at presentation are shown in **Fig. 1A**. On imaging evaluation at our hospital, breast ultrasonography revealed a 1.3-cm mass in the upper outer quadrant of the right breast (**Fig. 1B**). CT showed no evidence of distant metastasis (**Fig. 1C**), and breast MRI did not demonstrate apparent intraductal extension (**Fig. 1D**). The preoperative clinical stage was cT1N0M0, stage I.

**Fig. 2:**
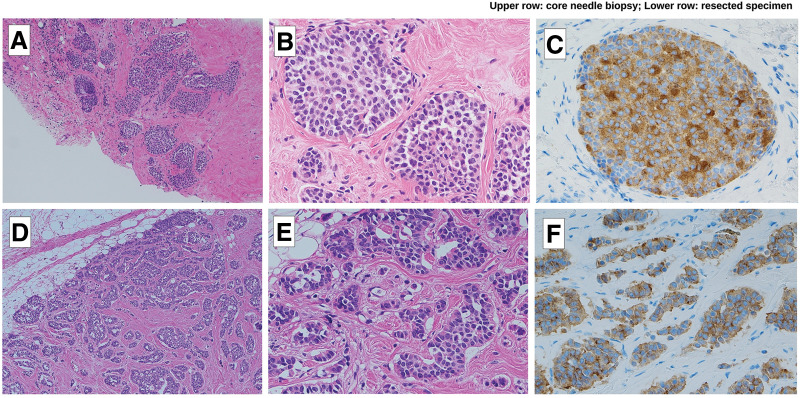
Histopathological findings of the CNB and resected specimen. Upper row: CNB specimen. Lower row: resected specimen. (**A**) Hematoxylin and eosin staining of the CNB specimen showing proliferation of relatively uniform epithelial cells arranged in small nests and solid structures. Original magnification, ×100. (**B**) High-power view of the CNB specimen showing tumor cells with relatively uniform cytological features. Original magnification, ×400. (**C**) Immunohistochemical staining of the CNB specimen showing positivity for synaptophysin. Original magnification, ×400. (**D**) Hematoxylin and eosin staining of the resected specimen showing invasive solid papillary carcinoma. Original magnification, ×100. (**E**) High-power view of the resected specimen showing atypical epithelial cells with enlarged nuclei proliferating in small nested and solid alveolar patterns. Original magnification, ×400. (**F**) Immunohistochemical staining of the resected specimen showing synaptophysin positivity, supporting neuroendocrine differentiation. Original magnification, ×400. CNB, core needle biopsy

Histological examination of the CNB specimen showed proliferation of relatively uniform epithelial cells arranged in small nests and solid structures (**Fig. 2A** and **2B**). The tumor cells were strongly positive for estrogen receptor and progesterone receptor, HER2-negative, and showed a low Ki-67 labeling index of 5%. Immunohistochemical staining for synaptophysin was positive (**Fig. 2C**). These findings suggested invasive solid papillary carcinoma with neuroendocrine differentiation, although distinction from invasive ductal carcinoma was challenging in the limited biopsy specimen. The patient underwent breast-conserving surgery and SLNB. The resected specimen showed invasive solid papillary carcinoma composed of atypical epithelial cells with enlarged nuclei proliferating in small nested and solid alveolar patterns (**Fig. 2D** and **2E**). Immunohistochemical findings were consistent with those of the biopsy specimen: estrogen receptor strongly positive, progesterone receptor strongly positive, HER2 negative, and Ki-67 labeling index 5%. Synaptophysin expression supported the diagnosis of solid papillary carcinoma with neuroendocrine differentiation (**Fig. 2F**).

Postoperative Oncotype DX Breast Recurrence Score testing (Exact Sciences, Madison, WI, USA) revealed an RS of 4. Based on the low genomic risk and hormone receptor-positive/HER2-negative phenotype, adjuvant chemotherapy was not administered. The patient received postoperative radiotherapy followed by adjuvant endocrine therapy with anastrozole 1 mg/day. She remains free of recurrence 1 year after surgery.

## DISCUSSION

Solid papillary carcinoma of the breast is a rare subtype of papillary breast carcinoma and is generally regarded as a low-grade neoplasm with favorable biological behavior.^9)^ It accounts for less than 1% of all breast cancers and predominantly affects elderly or postmenopausal women.^1,9)^ In this context, the present patient, who was 53 years old, represents a relatively young presentation for this histological subtype. Histologically, solid papillary carcinoma is characterized by well-circumscribed solid nodules composed of relatively uniform tumor cells arranged around delicate fibrovascular cores, and neuroendocrine differentiation is frequently observed.^2,10,11)^ Although many cases are considered *in situ* lesions, invasive solid papillary carcinoma has been recognized as a distinct clinicopathological entity with variable clinical behavior.^3,10,12,13)^

The central lesson of this case is the diagnostic difficulty encountered in the CNB specimen. At the previous hospital, the lesion was diagnosed as invasive ductal carcinoma. Pathological review at our institution revealed relatively uniform epithelial proliferation with small nested and solid growth patterns, strong estrogen receptor and progesterone receptor positivity, HER2 negativity, a low Ki-67 labeling index, and synaptophysin positivity. These findings supported invasive solid papillary carcinoma rather than conventional invasive ductal carcinoma. However, in limited biopsy material, the solid or nested architecture of invasive solid papillary carcinoma may closely resemble invasive ductal carcinoma of no special type, and assessment of papillary architecture and invasion can be challenging.^5–8,12,14)^

The clinical meaning of treatment de-escalation in the present case should be stated carefully. The tumor was cT1N0, strongly estrogen receptor-positive and progesterone receptor-positive, HER2-negative, and showed a Ki-67 labeling index of 5%. Therefore, adjuvant chemotherapy would probably not have been recommended even if the tumor had remained classified as invasive ductal carcinoma of no special type. Consistently, postoperative Oncotype DX testing revealed an RS of 4, supporting the omission of adjuvant chemotherapy, although the role of multigene assays in rare special histological subtypes should be interpreted cautiously.^15)^ Thus, accurate recognition of invasive solid papillary carcinoma did not directly avoid chemotherapy in this patient; rather, it provided a biologically appropriate framework for avoiding reflexive treatment escalation and for individualizing biopsy strategy, local treatment, axillary staging, and follow-up.

Preoperative diagnosis is increasingly important as local treatment options diversify. In Japan, RFA has recently become a minimally invasive local treatment option for selected patients with early-stage breast cancer, and reported eligibility criteria include histologically confirmed ductal carcinoma, no prior treatment, and a solitary localized tumor measuring 1.5 cm or less on preoperative imaging.^16)^ The present tumor measured 1.3 cm and was clinically node-negative; however, a special histological subtype such as invasive solid papillary carcinoma may fall outside the evidence base on which current RFA indications were established. Therefore, when this subtype is suspected preoperatively, the indication for RFA should incorporate histological certainty, imaging findings, radiologic–pathologic concordance, and multidisciplinary discussion.

A definitive preoperative diagnosis may also support surgical de-escalation, although caution is required. Regarding SLNB, solid papillary carcinoma generally shows an indolent course and a low frequency of nodal metastasis; nevertheless, axillary lymph node metastasis and distant metastasis have been reported in invasive cases.^10,13)^ Therefore, in a relatively young patient such as the present 53-year-old woman, a diagnosis of invasive solid papillary carcinoma alone would not currently justify the omission of SLNB. In future selected scenarios, particularly in elderly or frail patients with small, low-grade, clinically node-negative tumors and concordant imaging–pathological findings, this diagnosis may provide an additional rationale for considering the omission of axillary staging. For local excision, solid papillary carcinomas tend to form circumscribed nodules rather than extensive irregular intraductal components. A reliable preoperative diagnosis combined with MRI showing no extensive intraductal extension may support conservative margin planning, while margin decisions should still follow established principles for invasive carcinoma and associated intraductal disease.

Immunohistochemistry and adequate tissue sampling are essential for avoiding this diagnostic pitfall. Neuroendocrine markers, including synaptophysin and chromogranin, often support the diagnosis, although neuroendocrine differentiation is not specific to solid papillary carcinoma and is not required for the diagnosis.^11)^ Therefore, the diagnosis should integrate morphology, hormone receptor status, HER2 status, proliferation index, neuroendocrine differentiation, and myoepithelial markers when appropriate.^5,7,8,11,12)^ When CNB provides insufficient material or when morphology is discordant with the expected clinicopathological profile, additional sampling by VAB may be useful because it can obtain a larger tissue volume and facilitate evaluation of papillary architecture, fibrovascular cores, *in situ* and invasive components, myoepithelial markers, and neuroendocrine markers.^17)^

This report has several limitations. First, this is a single case report, and the clinical behavior of invasive solid papillary carcinoma cannot be generalized from 1 patient. Second, the follow-up period is currently limited to 1 year. Although the patient remains recurrence-free, longer follow-up is required, particularly because she was younger than the typical age distribution for solid papillary carcinoma and because invasive cases with nodal or distant metastasis have been reported.^10,13)^ Third, the role of multigene assays such as Oncotype DX in rare histological subtypes, including invasive solid papillary carcinoma, has not been fully established.^15)^ In this case, the RS should therefore be interpreted as supportive information rather than definitive evidence, although it was concordant with the tumor’s favorable clinicopathological profile.

## CONCLUSIONS

Invasive solid papillary carcinoma of the breast is a rare entity that can mimic invasive ductal carcinoma on CNB. The present case demonstrates that careful morphological assessment combined with immunohistochemical evaluation, including neuroendocrine markers, is essential for an accurate diagnosis. Correct recognition may help tailor treatment selection, including biopsy strategy, local therapy, and axillary staging, while avoiding inappropriate treatment escalation. However, because invasive cases with nodal or distant metastasis have been reported and the present follow-up period is limited to 1 year, long-term surveillance remains necessary.

## DECLARATIONS

### Availability of data and materials

All data generated or analyzed during this study are included in this published article.
